# A three-dimensional left atrial motion estimation from retrospective gated computed tomography: application in heart failure patients with atrial fibrillation

**DOI:** 10.3389/fcvm.2024.1359715

**Published:** 2024-03-26

**Authors:** Charles Sillett, Orod Razeghi, Angela W. C. Lee, Jose Alonso Solis Lemus, Caroline Roney, Carlo Mannina, Felicity de Vere, Kiruthika Ananthan, Daniel B. Ennis, Ulrike Haberland, Hao Xu, Alistair Young, Christopher A. Rinaldi, Ronak Rajani, Steven A. Niederer

**Affiliations:** ^1^ School of Biomedical Engineering and Imaging Sciences, King’s College London, London, United Kingdom; ^2^ National Heart and Lung Institute, Imperial College London, London, United Kingdom; ^3^ Department of Haematology, University of Cambridge, Cambridge, United Kingdom; ^4^ School of Engineering and Materials Science, Queen Mary University of London, London, United Kingdom; ^5^ Department of Medicine, Icahn School of Medicine at Mount Sinai, New York, NY, United States; ^6^ Department of Cardiology, Guy’s and St Thomas’ NHS Foundation Trust, London, United Kingdom; ^7^ Department of Radiology, Stanford University, Stanford, CA, United States; ^8^ Siemens Healthcare GmbH, Forchheim, Germany; ^9^ Turing Research and Innovation Cluster: Digital Twins, The Alan Turing Institute, London, United Kingdom

**Keywords:** left atrial strain, strain imaging, retrospective gated computed tomography, atrial fibrillation, heart failure

## Abstract

**Background:**

A reduced left atrial (LA) strain correlates with the presence of atrial fibrillation (AF). Conventional atrial strain analysis uses two-dimensional (2D) imaging, which is, however, limited by atrial foreshortening and an underestimation of through-plane motion. Retrospective gated computed tomography (RGCT) produces high-fidelity three-dimensional (3D) images of the cardiac anatomy throughout the cardiac cycle that can be used for estimating 3D mechanics. Its feasibility for LA strain measurement, however, is understudied.

**Aim:**

The aim of this study is to develop and apply a novel workflow to estimate 3D LA motion and calculate the strain from RGCT imaging. The utility of global and regional strains to separate heart failure in patients with reduced ejection fraction (HFrEF) with and without AF is investigated.

**Methods:**

A cohort of 30 HFrEF patients with (*n* = 9) and without (*n* = 21) AF underwent RGCT prior to cardiac resynchronisation therapy. The temporal sparse free form deformation image registration method was optimised for LA feature tracking in RGCT images and used to estimate 3D LA endocardial motion. The area and fibre reservoir strains were calculated over the LA body. Universal atrial coordinates and a human atrial fibre atlas enabled the regional strain calculation and the fibre strain calculation along the local myofibre orientation, respectively.

**Results:**

It was found that global reservoir strains were significantly reduced in the HFrEF + AF group patients compared with the HFrEF-only group patients (area strain: 11.2 ± 4.8% vs. 25.3 ± 12.6%, *P* = 0.001; fibre strain: 4.5 ± 2.0% vs. 15.2 ± 8.8%, *P* = 0.001), with HFrEF + AF patients having a greater regional reservoir strain dyssynchrony. All regional reservoir strains were reduced in the HFrEF + AF patient group, in whom the inferior wall strains exhibited the most significant differences. The global reservoir fibre strain and LA volume + posterior wall reservoir fibre strain exceeded LA volume alone and 2D global longitudinal strain (GLS) for AF classification (area-under-the-curve: global reservoir fibre strain: 0.94 ± 0.02, LA volume + posterior wall reservoir fibre strain: 0.95 ± 0.02, LA volume: 0.89 ± 0.03, 2D GLS: 0.90 ± 0.03).

**Conclusion:**

RGCT enables 3D LA motion estimation and strain calculation that outperforms 2D strain metrics and LA enlargement for AF classification. Differences in regional LA strain could reflect regional myocardial properties such as atrial fibrosis burden.

## 1 Introduction

A left atrial (LA) strain analysis provides insight into atrial mechanical dysfunction associated with the structural and functional remodelling that initiates and sustains atrial fibrillation (AF) (1). A reduced LA strain correlates with the prevalence of atrial fibrosis, a hallmark of the AF substrate, measured using late gadolinium enhancement (LGE) magnetic resonance imaging (MRI) (2–4), histology (5) and electroanatomic mapping data (6), as well as with AF presence (2), severity (3), and recurrence following catheter ablation (7).

Conventional LA strain analysis employs two-dimensional (2D) imaging techniques such as tissue Doppler imaging (TDI), speckle tracking echocardiography (STE), and cine MRI with feature tracking (FT) (8). This leads to atrial foreshortening and underestimates through-plane motion (9). A three-dimensional (3D) picture of LA motion is desirable to more accurately estimate LA mechanics and detect dysfunction (10). Whilst 3D STE and cine MRI imaging solutions exist, they remain limited by image quality and spatial resolution similar to or worse than typical LA wall thickness (0.6–3.0 mm) (11, 12).

An alternative imaging modality is retrospective gated computed tomography (RGCT), which uses electrocardiogram (ECG) gating to produce multiple computed tomography (CT) images over the cardiac cycle. RGCT can be indicated for pre-procedural planning in cardiac resynchronisation therapy (CRT) and is clinically indicated in patients undergoing transcatheter aortic valve replacement (TAVR) for valvular sizing (13). The high CT spatial resolution enables RGCT to produce high-fidelity 4D (3D + time) images of the complex LA anatomy (14). Therefore, in this study, we hypothesise that RGCT with FT can be used to provide a detailed regional estimation of 3D LA mechanics.

RGCT has previously been applied to 3D LV motion estimation and regional systolic strain measurement. Regional LV myocardial shortening has been measured using non-rigid surface registration between LV endocardial surfaces from end-diastole (ED) to end-systole (ES) and applied to strain measurement (15) and mechanical activation time estimation (16). LV systolic strain synchrony, measured using the temporal sparse free form deformation (TSFFD) non-rigid image registration method (17), found more dyssynchronous LV mechanics in patients with heart failure (HF) than in control subjects (18).

In contrast to that for the LV, there is a relative dearth of studies that investigate the feasibility of using RGCT to estimate 3D LA mechanics. 2D LA strain using RGCT has previously been reported (19, 20) and compared with 2D STE (21). Cine MRI with feature tracking has been used to estimate 3D LA mechanics (22, 23); however, images with large slice thickness and in-plane resolution similar to LA wall thickness were used for this purpose, which is likely to impact imaging fidelity, and the accuracy of 3D feature tracking was not reported. The accuracy of material point tracking in RGCT using 4D motion estimation software and implanted glass beads was reported in 8 swine atria (24); however, this was not extended to human subjects.

In this study, we present the results of the estimated 3D LA motion and calculated the strain using the TSFFD method to track LA features in RGCT images in a cohort of patients with heart failure with reduced ejection fraction (HFrEF). The TSFFD image registration method was chosen since it enables strain calculation using only a single segmentation of the LA blood pool at the ED time frame, and has previously been optimised using manual landmarks for LV motion estimation (18). In this study, the TSFFD configuration was optimised specifically for LA FT in RGCT. The reservoir strain, corresponding to peak atrial expansion, was quantified both globally and regionally using the universal atrial coordinate (UAC) system and compared between patients with and without AF. Calculated area and fibre strains quantified the 3D LA motion. A human atrial fibre atlas enabled fibre strain calculation, which provides the change in length parallel to the local myofibre orientation.

We hypothesised that patients with AF would have a reduced LA reservoir strain as they are likely to have greater atrial remodelling, and that the best-performing regional strain for AF classification could signify candidate locations for preferential fibrosis deposition. In addition, we tested the hypothesis that the strain derived from the estimated 3D motion exceeds the strain derived from the estimated 2D motion from two- and four-chamber views for AF classification. Finally, we investigated differences in the regional dyssynchrony and heterogeneity of LA expansion between patient groups.

## 2 Methods

**Figure 1:**
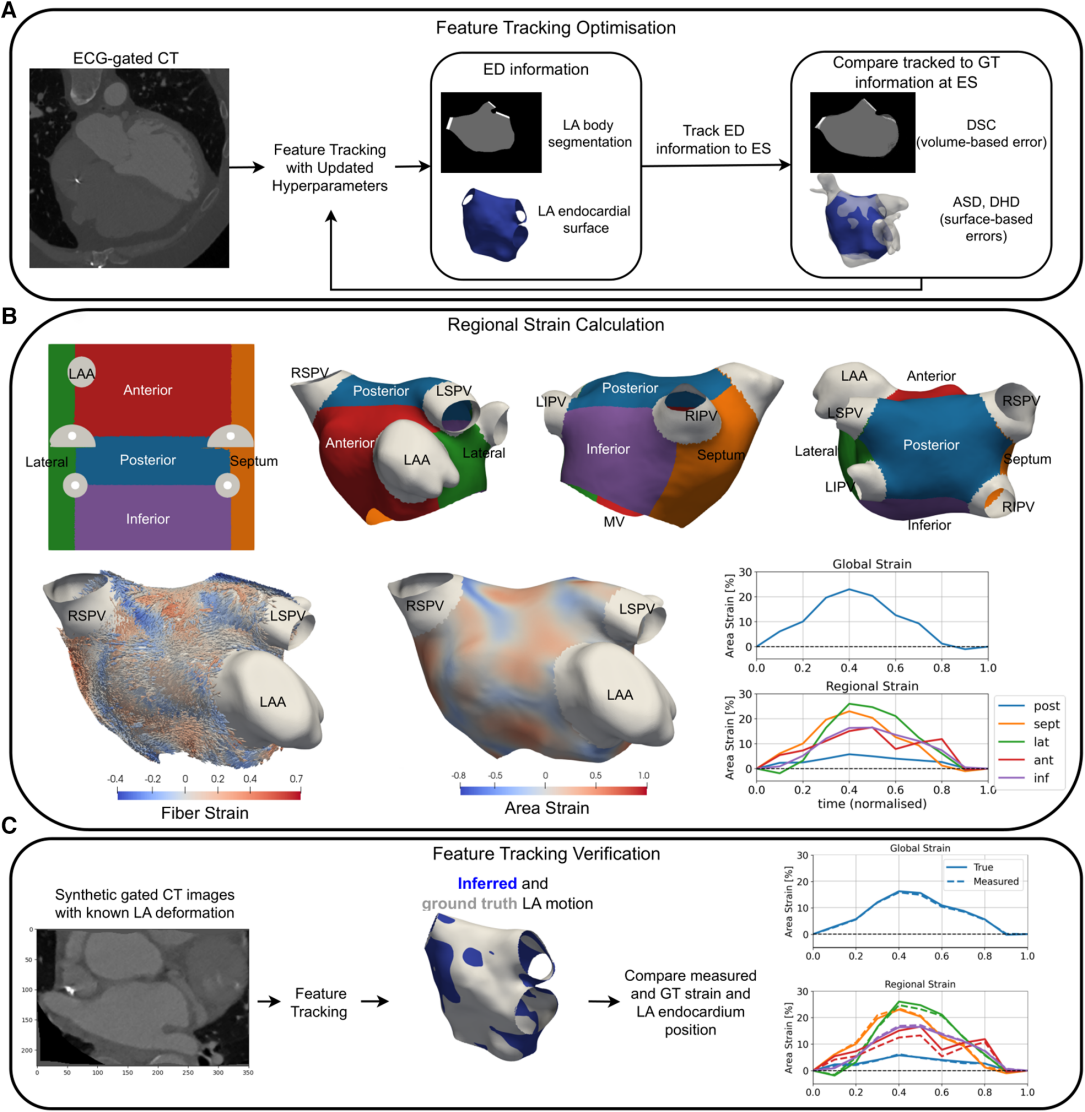
An overview of a pipeline for optimisation (**A**), the application (**B**), and verification (**C**) of LA strain measurement using RGCT. (**A**) Feature tracking software hyperparameters were optimised to minimise volume-based errors using the DSC and surface-based errors using the DHD and ASD of the LA endocardium at ES. (**B**) Universal atrial coordinates were used to separate the LA body into five regions (top row). Area and fibre strain maps at ES are overlaid on a sample case anatomy. Global and regional area strain transients are shown for a HFrEF-only case. (**C**) Tracking of LA features was verified using synthetic data with ground truth LA motion, where material point tracking and strain errors were calculated. GT, ground truth; LSPV, left superior pulmonary vein; LIPV, left inferior pulmonary vein; RSPV, right superior pulmonary vein; RIPV, right inferior pulmonary vein.

An overview of the methods is outlined in Figure 1. Full details can be found in the Supplementary material.

### 2.1 Patient cohort

The study complied with the Declaration of Helsinki and the protocol was approved by the regional ethics committees. Each patient provided written informed consent, and images were anonymised prior to analysis. Between 2014 and 2018, we recruited 30 HFrEF patients with a pre-existing pacemaker/implantable cardioverter-defibrillator, left bundle branch block (LBBB) (56.7%), persistent HF symptoms on optimal medical therapy, and a left ventricular ejection fraction (LVEF) <50%. A retrospective ECG-gated CT was indicated for CRT upgrade planning. AF presence and type were based on the most recent device check prior to the date of the CT. LVEF, left atrial emptying fraction (LAEF), and LA and LV end-diastolic volume (EDV) were calculated from the gated CT images.

### 2.2 Imaging protocol

CT images were acquired using a Siemens SOMATOM Force Dual Source scanner (Siemens Healthcare GmbH, Forchheim, Germany). A total of 80 ml of intravenous contrast agent (Omnipaque; GE Healthcare, Princeton, NJ, USA) was injected via a power injector into the antecubital vein. Helical scanning was performed with a single breath-hold technique after a 10–12 s delay. The scanning parameters included a heart rate–dependent pitch of 0.15–0.35 and a gantry rotation time of 250 ms. Tube voltage (100/110/120 kV) and tube current were adapted depending on patient size by the automatic dose modulation CareDose4D with reference values of 100 kV and 300 mAs. Retrospective ECG gating was used to generate either 10 or 20 CT datasets in 10% or 5% increments per cardiac cycle, respectively. The in-plane isotropic image resolution ranged between 0.32 and 0.49 mm and slice thickness ranged between 0.4 and 1.2 mm. All images were acquired in right ventricular (RV) pacing and sinus rhythm (SR).

### 2.3 Patient-specific model creation

LA endocardial surface models were created from the ED CT frame segmentation using CemrgApp (25–27). UACs were calculated on each anatomy (28) and an average endocardial fibre architecture from a dataset of *ex vivo* human hearts was mapped to each patient-specific model (29, 30). The LA body was separated into five regions (posterior, septum, lateral, anterior, and inferior walls) using the UACs (Figure 1B).

### 2.4 Feature tracking

This section provides details of the application, optimisation, and verification of the TSFFD method (17) for LA feature tracking in RGCT images. Further details can be found in the Supplementary material.

#### 2.4.1 Temporal sparse free form deformation method

The TSFFD method was utilised in this study as it is based on the widely used free form deformation method (31) with additional control point (CP) sparsity and temporal cyclicity constraints that make it appropriate for the estimation of physiologically realistic cardiac motion. Furthermore, TSFFD has previously been validated for LV feature tracking in RGCT images using expert-chosen landmarks (18) that served as a baseline configuration prior to the LA chamber–specific optimisation.

Briefly, the TSFFD method tracks the borders of the LA endocardium using image similarity matching with a regularisation that encourages smooth motion in both space and time (see Supplementary Videos S1–4 for dynamic visualisations of the measured 3D CT-derived LA motion). Feature tracking was performed using 10 RGCT frames at 10% reconstructions of the R-R interval, and non-rigid image registration of all later CT frames with the initial ED frame was performed simultaneously. This facilitated temporal regularisation for feature tracking. Multiple levels of CPs with increasing spatial resolution were used, and CP displacements were parameterised with cubic B-splines in both space and time to encourage spatially and temporally smooth displacements. The sparsity constraint encouraged sparsely activated CPs. We found using the normalized mutual information image similarity metric gave qualitatively more accurate image registration than using the mean squared error.

#### 2.4.2 LA chamber–specific optimisation

The TSFFD method has two important hyperparameters: the bending energy (BE) and sparsity weight (SW), which parametrise the spatial smoothness and sparsity constraint of the displacement fields, respectively. A grid search through 63 different (SW, BE) combinations was iterated through at a coarse and a fine multi-level CP set, which are outlined in the Supplementary material. The (SW, BE) values iterated through were centred on the optimal combination used for LV feature tracking in RGCT images found in Razeghi et al. (18). In addition, a value of SW of 0.0 was used to assess the sparsity constraint.

Segmentations and surface meshes of the LA body at ES (*t* = 40%) served as the ground truth to assess LA feature tracking accuracy in all 30 cases. The ED-ES pair was chosen since it involves maximal LA expansion during the atrial reservoir phase and therefore is likely to yield the largest errors in LA feature tracking accuracy. Feature tracking accuracy was assessed using the average surface distance (ASD), directed Hausdorff distance (DHD), and Dice score coefficient (DSC) between tracked and ground truth surface meshes and segmentations as previously described in (32). Equal weighting was used to combine the ASD, DHD, and DSC errors for identifying the optimal (SW, BE) combination, and five-fold cross-validation was used to ensure that hyperparameter optimisation was not specific to the cases used to quantify feature tracking accuracy.

#### 2.4.3 Feature tracking verification

We verified our feature tracking method using synthetic-gated CT images in which the underlying LA motion was known. Synthetic RGCT images were created by applying displacement fields output from the TSFFD method (17) to the ED CT image for all 30 cases. The synthetic images were then re-registered to re-calculate LA body displacements and strains. This enabled us to compare ground truth and inferred endocardium positions, strain transients, and reservoir strain measurements.

### 2.5 Strain measurement

#### 2.5.1 Strain calculation

Area and fibre strains were calculated with respect to the LA endocardium at ED that coincided with the R-wave on the ECG. Area and fibre strains were defined as the percentage area change and percentage length change along the local endocardial myofibre orientation of a local element, respectively.

#### 2.5.2 Reservoir strain and regional strain

The reservoir strain was measured by taking the maximum minus minimum strain values over the cardiac cycle to avoid errors from possible ECG mis-triggering. Global and regional strains were calculated by taking the average elemental strain over the entire LA body surface and within each region, respectively.

#### 2.5.3 Two-dimensional strain measurement

To compare the LA strain derived from 2D and 3D LA motions, 2D global longitudinal strain (GLS) was measured from two- and four-chamber views extracted from the RGCT images. Manual landmarks defined the two-dimensional planes, and the TSFFD method was used to track LA features in 2D. The strain was calculated as the percentage change in LA body contour length, excluding the mitral valve (MV), pulmonary veins (PVs), and left atrial appendage (LAA), and the reservoir strain was measured by taking the maximum minus minimum strain values. The mean reservoir strain across both the two- and the four- chamber views yielded 2D GLS.

### 2.6 Statistical analysis

Continuous variables are reported as mean ± standard deviation. Categorical variables are presented as frequencies and percentages. Differences between patient groups were evaluated using a two-sided *t*-test and the chi-squared test. Statistically significant results were taken as *P* < 0.05. Receiver operator characteristic (ROC) analysis used raw data values for different variables for binary AF classification.

## 3 Results

### 3.1 Cohort properties

The demographics are outlined in Table 1. Medications are provided in the Supplementary material. Strain measurement was possible in all patients. All patients were in SR during imaging to ensure that differences in the measured LA strain resulted from differences in LA myocardial properties and not from rhythm during imaging.

**Table 1:** Demographics table.

	HFrEF-only (*n* = 21)	HFrEF + AF (*n* = 9)	*P*-value
Age (years)	66.5 ± 14.2	71.4 ± 7.4	0.34
Sex (male), *n* (%)	17 (81.0)	8 (89.0)	0.61
LVEF (%)	34.5 ± 11.2	30.6 ± 7.0	0.35
LAEF (%)	32.3 ± 14.7	10.9 ± 4	<0.001
LV EDV (ml)	273.9 ± 85.5	271.9 ± 83.0	0.95
LA EDV (ml)	96.0 ± 36.9	163.1 ± 45.1	<0.001
QRS duration (ms)	159.1 ± 21.5	158.6 ± 28.9	0.96
LBBB, *n* (%)	14 (66.7)	3 (33.3)	0.10
AF type			
Paroxysmal	0	3	
Persistent	0	6	
NYHA class			
II	7	5	0.46
III	14	4	0.46
Device			
Single PPM	2	2	0.73
Dual PPM	10	2	0.37
Dual ICD	3	4	0.19
CRT-D	6	1	0.57
Prior ablation?	1	3	0.13
Mitral regurgitation severity			
None	2	0	0.87
Trace	4	1	1.0
Trivial	3	2	1.0
Mild	7	4	0.87
Moderate	2	1	1.0
Severe	3	1	1.0

LV EDV, left ventricular end-diastolic volume; NYHA, New York Heart Association; PPM, permanent pacemaker; ICD, implantable cardioverter-defibrillator; CRT-D, CRT defibrillator.

### 3.2 Feature tracking verification

Our optimised feature tracking method reproduced LA endocardial surface coordinates at ES with a root mean square error (RMSE) of 0.78 ± 0.60 mm and 0.40 ± 0.13 mm across the HFrEF-only and HFrEF + AF patient groups, respectively. This was of the same order of magnitude of the image resolution, which suggests that our method predicts LA endocardial material point position within two image voxels.

### 3.3 Regional reservoir strains

**Figure 2:**
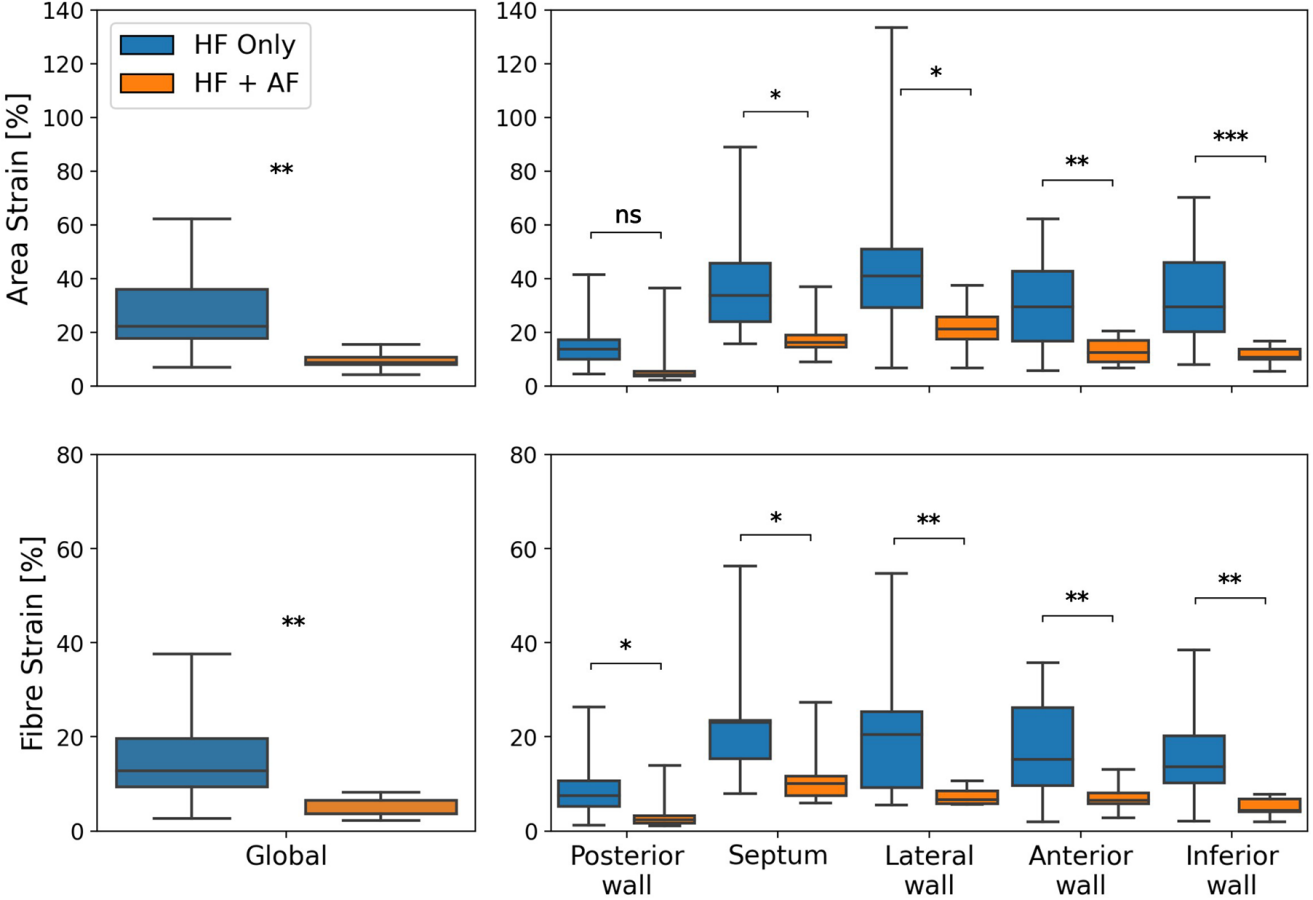
A comparison of global (left column) and regional (right column) reservoir strains between HFrEF-only and HFrEF + AF patients. Area strains (top row) and fibre strains (bottom row) are shown. *P*-value legend, ns: not significant; *P < 0.05; **P < 0.01; ***P < 0.001.

To test the impact of AF presence on LA reservoir strain magnitude, global and regional reservoir strains were compared between the HFrEF + AF and the HFrEF-only groups (Figure 2). It was found that the global reservoir strains were significantly decreased in the HFrEF + AF group across both the area and the fibre strain metrics (*P* = 0.001 for both). Regionally, the HFrEF + AF group exhibited a significantly (*P* < 0.05) smaller reservoir strain across all regions except for the posterior wall area strains.

**Figure 3:**
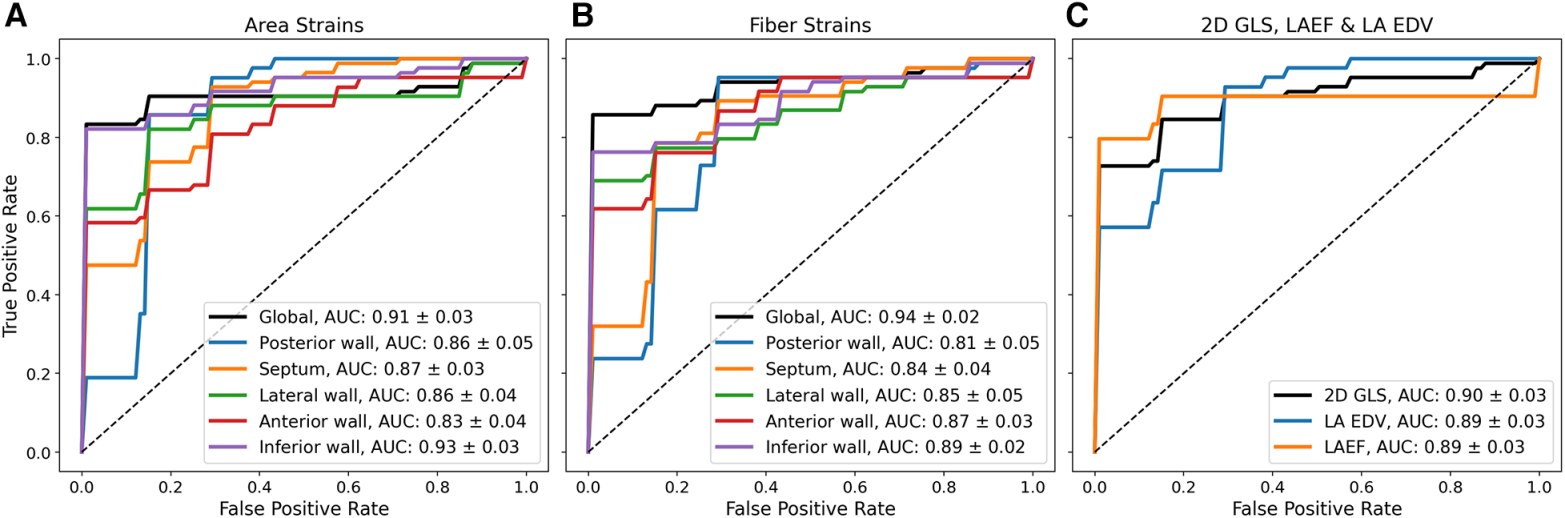
Mean ROC curves from five-fold cross-validation using different biomarkers for binary classification between HFrEF + AF and HFrEF-only patients. Mean ROC curves and AUC values (mean ± standard deviation) are shown using (**A**) reservoir area strains (**B**), reservoir fibre strains, and (**C**) 2D GLS; LAEF and LA EDV for binary classification.

To test whether the reservoir strain stratified between groups, a ROC analysis with five-fold cross-validation was performed. Figure 3 shows mean ROC curves and area-under-the-curve (AUC) values. The global reservoir strain identified AF with high accuracy (AUC, area strain: 0.91 ± 0.03; fibre strain: 0.94 ± 0.02). The inferior wall provided the optimal regional reservoir strains for AF classification (AUC, area strain: 0.93 ± 0.03; fibre strain: 0.89 ± 0.02). The global reservoir fibre strains exceeded LAEF, LA EDV, and 2D GLS for AF classification (AUC, global fibre strain: 0.94 ± 0.02, LAEF: 0.89 ± 0.03, LA EDV: 0.89 ± 0.03, 2D GLS: 0.90 ± 0.03). To investigate the additive value of LA functional information upon an enlarged LA volume for AF classification, the ROC analysis was performed with all functional parameters combined with LA EDV. All biomarkers were normalised prior to combination. The combination of the posterior wall reservoir fibre strains with LA EDV provided the greatest improvement in the performance of classification, outperforming LA EDV + LAEF and LA EDV + 2D GLS (AUC, LA EDV + posterior wall fibre strain: 0.95 ± 0.02; LA EDV + LAEF: 0.92 ± 0.02; LA EDV + 2D GLS: 0.92 ± 0.02).

**Figure 4:**
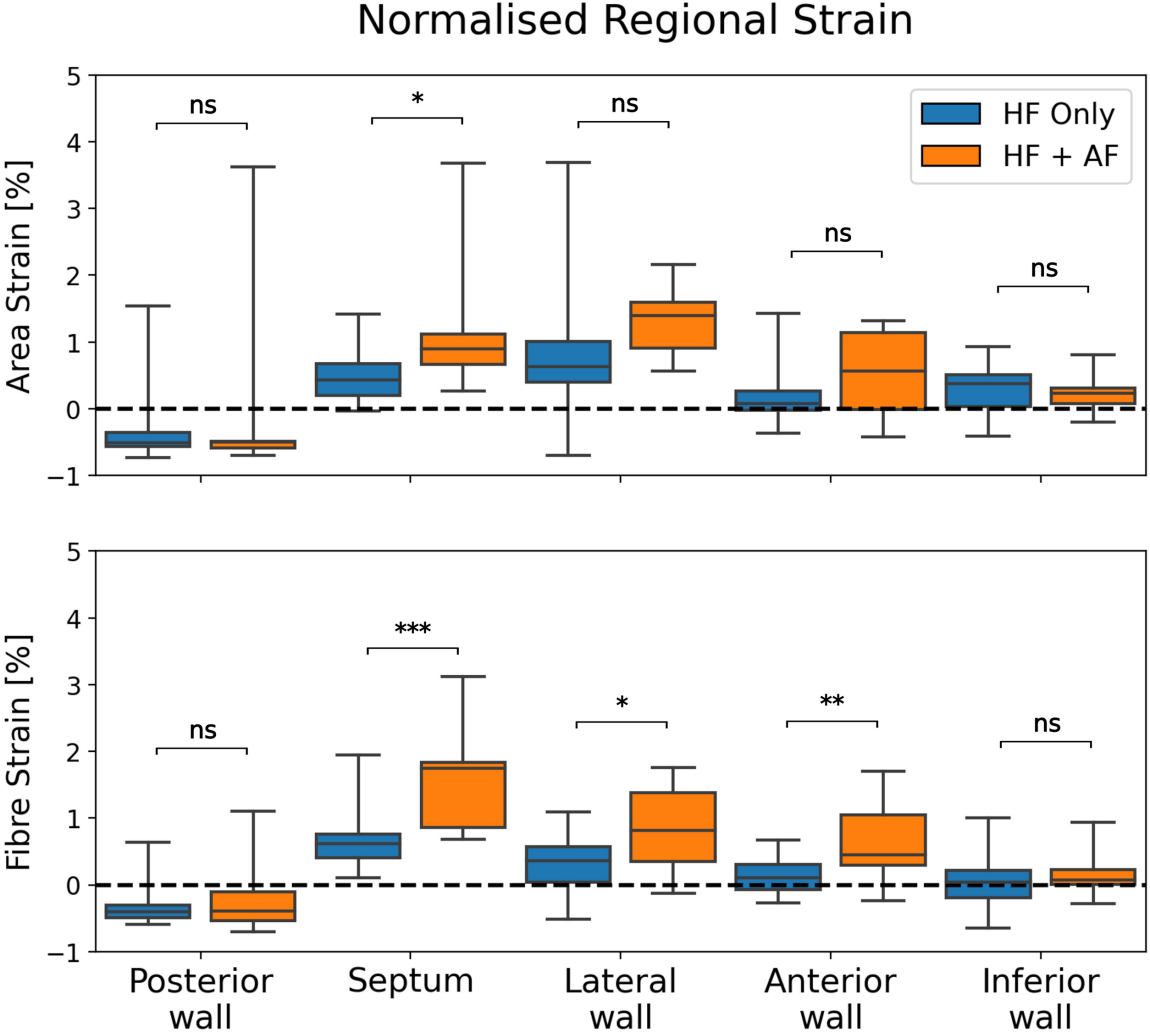
A comparison of normalised regional reservoir strains between groups. Regional reservoir strains were normalised with respect to the global reservoir strain for each patient. This provided an adjustment for global strain differences and enabled a comparison of the spatial heterogeneity of reservoir strain magnitude between groups. *P*-value legend, ns: not significant; *P < 0.05; **P < 0.01; ***P < 0.001.

The regional strain analysis showed a spatially heterogeneous reservoir strain magnitude across the LA body within both groups (Figure 2). The posterior wall adjacent to the PVs exhibited the smallest strains (area strain: 11.4 ± 8.5%; fibre strain: 8.1 ± 6.7%). The regions that exhibited the largest strains were the septum (area strain: 29.2 ± 13.7%; fibre strain: 19.8 ± 12.7%) and lateral wall (area strain: 32.8 ± 14.8%; fibre strain: 16.3 ± 12.6%). To investigate whether the spatial heterogeneity of reservoir strain magnitude changed in the presence of AF, regional reservoir strain measurements were normalised with respect to the global reservoir strain for each patient and compared between the groups (Figure 4). Negative and positive normalised regional strain values indicated lower and higher regional strains compared with the global average strain, respectively. When the regional strains were normalised, the relative depression of the regional reservoir strain in the HFrEF + AF vs. the HFrEF-only group was largely removed.

### 3.4 Regional dyssynchrony in reservoir strains

**Figure 5:**
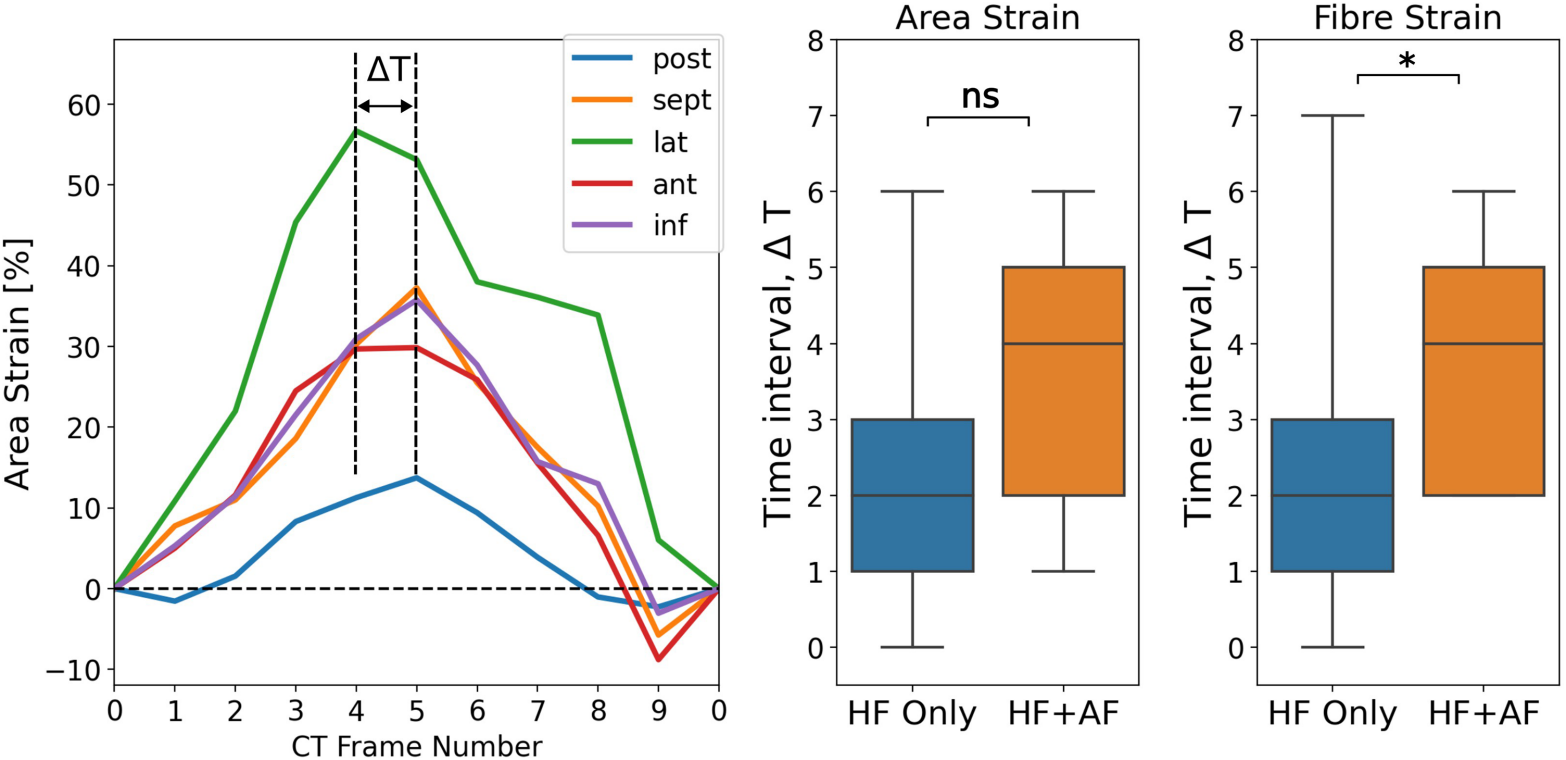
Measuring LA regional reservoir strain dyssynchrony by calculating the time interval, ΔT, over which regional strain transients peak. The units for ΔT are the number of CT time frames. ΔT was calculated for both strain metrics and compared between groups. *P*-value legend, ns: not significant; *P < 0.05; **P < 0.01; ***P < 0.001.

The spatial synchrony of the LA reservoir strain was measured using the range of time frames over which regional strain transients peaked. Figure 5 depicts the calculation of the time interval, ΔT, over which regional strain transients peak and shows a comparison of ΔT between the groups. ΔT was greater in the HFrEF + AF group across both strain metrics (area strain: 3.4 ± 1.7 vs. 2.3 ± 1.6, *P* = 0.10; fibre strain: 3.8 ± 1.5 vs. 2.4 ± 1.5, *P* = 0.03). To investigate whether dyssynchrony in the LA reservoir strain was related to LV contractile dyssynchrony, QRS duration (QRSd) was compared with ΔT. We found no strong correlation (absolute *r* < 0.15) between QRSd and ΔT across strain metrics.

## 4 Discussion

This study presents a novel workflow to estimate 3D LA motion and calculate strain from RGCT images. We found that global and regional reservoir strains were reduced in the HFrEF + AF patient group compared with the HFrEF-only patient group, where the inferior wall reservoir strains exhibited the most significant differences between the groups. The regional reservoir strain was more dyssynchronous in the presence of AF, whilst regional strain magnitude heterogeneity was conserved. The global reservoir fibre strain and LA volume combined with the posterior wall reservoir fibre strain exceeded LA volume alone and 2D GLS for AF classification.

### 4.1 Comparison with other modalities

A 3D picture of atrial motion could provide greater insights than 2D methods for detecting mechanical dysfunction. Previous studies evaluated 3D LA motion using cine MRI with a 2 mm isotropic resolution and a 1.4 × 1.4 × 10 mm^3^ resolution (22, 23) that are of the same, or greater, order of magnitude than typical LA wall thickness (12). In this study, RGCT with an in-plane isotropic resolution between 0.32 and 0.49 mm and slice thickness between 0.4 and 1.2 mm was used, which is likely to yield higher-fidelity images of the LA anatomy and aid accurate 3D feature tracking.

### 4.2 Feature tracking accuracy

Few studies have reported about the factor of uncertainty in 3D LA feature tracking. Ashikaga et al. reported a mean error of 0.76 mm between true and tracked implanted glass beads coordinates in swine atria using gated CT with feature tracking (24). In this study, *in silico* synthetic CT images with known LA motion verified material point tracking. We found a median RMSE of less than 0.8 mm in LA endocardial surface coordinates and an absolute error of less than 3% in reservoir strain measurements across the entire cohort. This suggests that our method was able to predict the LA strain and position accurately.

### 4.3 Strain differences in HFrEF patients with atrial fibrillation

There were no significant differences in the LVEF between the HFrEF + AF and the HFrEF-only groups, which suggests that LA reservoir strain differences were not driven by differences in LV systolic function. Reduced global and regional reservoir strains were found in the presence of AF, which is consistent with the findings in the literature (2, 3). Regional reservoir strain magnitude was spatially heterogeneous, which is consistent with that of previous studies that used 2D echocardiography and MRI (33, 34).

Area and fibre strains derived from the estimated 3D motion from the original RGCT images exceeded the GLS derived from the estimated 2D motion from the two- and four-chamber views for AF classification. This suggests that a 3D picture of LA motion encapsulates more information than 2D techniques for detecting dysfunction related to AF presence.

The depression in reservoir strain magnitude in the HFrEF + AF group ceased when regional strains were normalised to each patient's global reservoir strain. This suggested that the spatial heterogeneity of the atrial reservoir strain was preserved in the presence of AF. Therefore, we did not observe differences in strain heterogeneity that would be expected if fibrosis was preferentially present in one region. This could be explained by the comparison between HFrEF patients with and without AF. The HFrEF-only group probably had a non-zero fibrosis burden because of their pathology (35). Furthermore, fibrosis location may not be consistent across the HFrEF + AF group. Regional fibrosis burden location is variable (36), which may make a group-level comparison unsuitable to measure a regionally inhibited LA strain caused by fibrosis for a given patient. To provide stronger evidence linking regionally reduced strain with fibrosis, increased patient numbers or a comparison of the regional strain with a regional voltage or LGE MRI measurement may be necessary.

The ROC analysis revealed that there was an improvement in the performance of AF classification when combining structural with functional LA parameters. The posterior wall reservoir fibre strains improved the classification performance the most when combining a single functional parameter with LA EDV. This suggested that the posterior wall fibre strains contained the maximum amount of information for predicting AF presence that was not already captured by LA enlargement. Interestingly, this was not replicated when combining LA EDV with the posterior wall area strains, which suggests that directional information of LA deformation in the posterior wall is important for a more comprehensive analysis to identify AF.

The HFrEF + AF group of patients exhibited a greater dyssynchrony in regional reservoir strains. This was unlikely to be driven by dyssynchrony in LV systolic function because we observed a weak (*r* < 0.15) correlation between QRSd and ΔT. Increased heterogeneity in LA myocardial properties could explain this observation, which might be consistent with a greater regional fibrosis burden in the HFrEF + AF group and evidence of a non-uniform spatial distribution of atrial fibrosis (36).

### 4.4 Atrial fibre strains

To the best of our knowledge, this study presents the first estimation of LA fibre strains. We hypothesised that fibre strain may provide a more sensitive marker for differences in LA mechanical function between HFrEF + AF vs. HFrEF-only patients as fibre strain has previously been reported to be homogeneous throughout the LV wall (37, 38). We found that the global reservoir fibre strain gave a greater AUC value for AF classification than the global reservoir area strain did; however, the regional reservoir strains did not replicate this finding. It is possible that the interplay between deformation and myofibre orientation differs in the LA vs. the LV because of its smaller scale, the complex atrial anatomy, and the complex boundary loading from the mitral valve, pulmonary veins, and right atrium. We note that an average myofibre atlas was used, which may not reflect the fibre orientation of each individual patient. Measurement of patient-specific atrial fibre architecture *in vivo*, however, remains impractical.

### 4.5 Clinical perspective

We acknowledge that RGCT is limited in its indication because of the high x-ray exposure for patients. However, our study demonstrates the feasibility of using RGCT to provide a 3D estimation of LA motion, which has previously been understudied in comparison with the LV, and the additional information this captures that surpasses 2D strain and LA volume measurement for identifying AF presence. Our workflow can be applied for patients who are clinically indicated for RGCT imaging, such as TAVR patients for valvular sizing throughout the cardiac cycle and CRT patients for pre-procedural planning for device implantation. Our workflow represents efforts to maximise the utility of available imaging data for these cohorts and enables a highly detailed evaluation of regional LA mechanical function that is relevant to patients suffering from, or at risk of developing, AF and mitral regurgitation. Furthermore, our method offers a potential alternative regional assessment of LA cardiomyopathy for patients with implanted devices that make LGE MRI assessment difficult. However, further work is required to investigate the link between regional fibrosis burden and regional RGCT-derived LA strain.

### 4.6 Limitations

This retrospective, single-centre study imaged a small cohort of HFrEF patients who were indicated for RGCT for CRT planning. Larger multi-centre studies that are indicated for RGCT should be evaluated to verify our findings. In this study, only 10 CT frames from RGCT were used in the feature tracking to estimate LA motion to reduce computational cost and processing times. Whilst it was likely that 10 CT frames accurately captured the point of maximum LA expansion, corresponding to LA reservoir strain measurement, the reservoir strain using all 20 CT frames in available patients should be evaluated for comparison in future studies. In this study, strains were compared between HFrEF-only and HFrEF + AF patients who were likely to have a higher fibrosis burden. In future studies, regional data from voltage maps or LGE MRI should be compared with strains from our workflow in order to comment on the quantitative relationship between fibrosis and CT-derived strains.

### 4.7 Conclusions

We developed, tested, and applied a novel workflow to estimate 3D LA motion and calculate the strain from RGCT images in a cohort of HFrEF patients with and without AF. Our method enabled LA fibre strain estimation and a regional analysis of LA reservoir strain, which could identify differences in LA function in HFrEF patients with AF.

## Data Availability

The data analysed in this study are subject to the following licenses/restrictions: Data were collected as part of two clinical trials. Requests to access these datasets should be directed to charles.sillett@kcl.ac.uk.
