# Supplementary material for: A three-dimensional left atrial motion estimation from retrospective gated computed tomography: application in heart failure patients with atrial fibrillation
Source: Front Cardiovasc Med. 2024 Mar 26;11:1359715. doi: 10.3389/fcvm.2024.1359715 (PMC11002108; doi:10.3389/fcvm.2024.1359715)
Supplement: Supplementary file 5 [file Datasheet1.pdf]

Supplementary Material

1 Patient-specific Model Creation

Patient-specific models of the left atrial (LA) endocardium were created using the steps below.

1.1 Segmentation and Meshing

A convolutional neural network (CNN) was used to automatically generate a 3D whole-heart multilabel segmentation from the end-diastolic CT time frame [27]. The body of the left atrium (LA), left atrial appendage (LAA) and the left and right pulmonary veins (PVs) were extracted from the multilabel segmentation as a single LA label, as shown in Figure 1A. Segmentations that leaked into the ascending aorta, or where the LAA and left superior PV (LSPV) joined were manually corrected using CemrgApp [25]. Cases where the CNN failed to generate an output were segmented using a semi-automatic region-growing tool and 3D interpolation in CemrgApp.

LA endocardial surface meshes were generated from the LA segmentations using the Medical Image Registration Toolkit (MIRTK), as shown in Figure 1B. Surface meshes were post-processed using CemrgApp which gave an output depicted in Figure 2A. The sleeve extent of the PVs and LAA were labelled semi-automatically using clippers that were generated using a Voronoi diagram representation of the LA. The PVs and mitral valve (MV) were clipped using spherical clippers defined by the user.

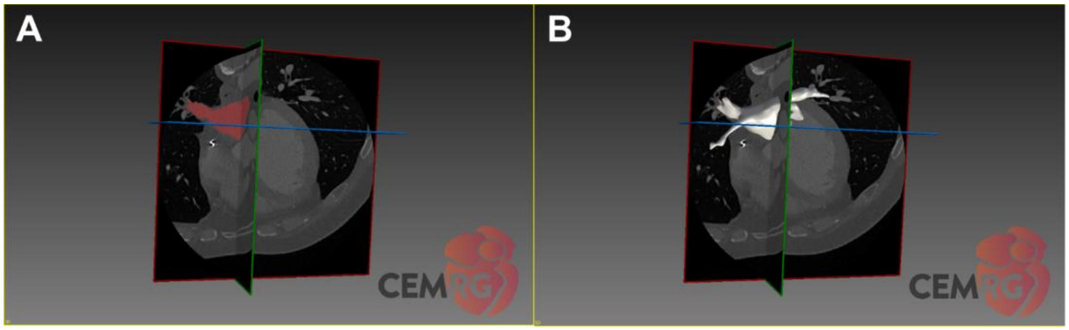

Supplementary Figure 1. Segmentation and surface meshing of the left atrium (LA) from end-diastolic CT image. **A:** The output segmentation of the LA body, pulmonary veins (PVs) and left atrial appendage (LAA) from a CNN. **B:** Surface mesh of the LA endocardium extracted from the LA body segmentation.

1.2 Universal Atrial Coordinates and Fiber Fields

Universal atrial coordinates (UACs) were registered to each patient's anatomical model by manually selecting 4 landmarks on the endocardium that were used to define the orthogonal axes of the UACs. The landmarks were selected as outlined in Roney et al [28] and were defined as: the LSPV/posterior wall boundary, RSPV/posterior boundary, fossa ovalis and left atrial appendage.

Average endocardial fiber fields from a dataset of seven ex vivo human hearts imaged using diffusion tensor magnetic resonance imaging [29] (DT-MRI) were appended to each model using the registered UACs, shown in Figure 2B. This provided a myofiber vector direction for each element on the model.

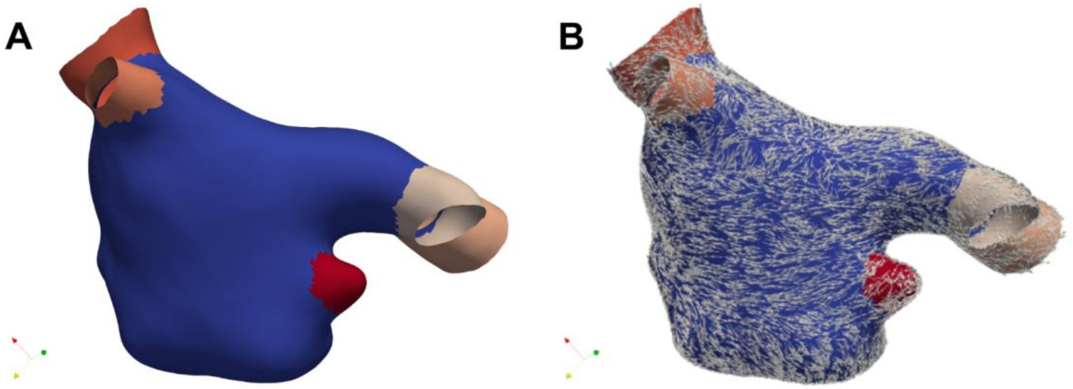

Supplementary Figure 2. Refinement of the surface mesh of the LA endocardium and registration of a human atrial fiber atlas. **A:** The LA endocardium surface was refined in CemrgApp: the LAA and all four PVs and their extents were labelled semi-automatically. The mitral valve (MV) and PVs were manually clipped using spheres. **B:** An atrial fiber atlas from a human ex vivo dataset was appended to each model using the Universal Atrial Coordinates (UACs). The UAC registration enabled each surface elements to be assigned an estimated endocardial myofiber orientation, denoted by the grey arrowed glyphs.

1.3 Regional Separation of the LA Body using UACs

The LA body was separated into 5 different regions: posterior wall, septum, lateral wall, anterior wall and inferior wall using the UACs. The pulmonary vein positions were used as landmarks to create the borders between the 5 regions, as depicted in Figure 3.

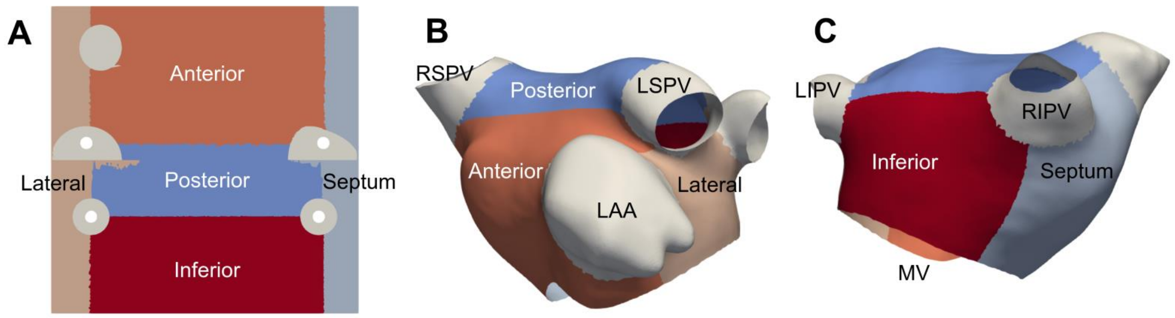

Supplementary Figure 3. Separation of the LA body in 5 regions (posterior, septum, lateral, anterior and inferior walls) using the UACs. **A:** The pulmonary veins (PVs) were used as landmarks to delineate the LA body into 5 regions, denoted by different colours. The PVs and LAA area shaded light gray to show these regions were omitted from the strain calculations. This ensured only strains from the LA body contributed to strain measurement. **B & C:** The 5 regions viewed on the LA anatomy. Abbreviations: LAA – left atrial appendage, LSPV – left superior pulmonary vein, LIPV – left inferior pulmonary vein, RSPV – right superior pulmonary vein, RIPV – right inferior pulmonary vein, MV – mitral valve.

2 Imaging Data

Retrospective electrocardiogram (ECG)-gated computed tomography (CT) imaging was indicated for each patient prior to cardiac resynchronization therapy (CRT). Ten or twenty frame CT images, at 10% or 5% intervals between the R-R interval and starting from the R-wave, were created for each patient. In this study, ten CT image frames were used to measure LA motion and strain. The dimension of images varied between patients, however the majority of cases had in-plane shape of 512 x 512 voxels with a range of 0.32-0.49 mm isotropic resolution, and a range of 94-399 slices with a through-plane thickness of 0.4-1.2 mm.

3 Feature Tracking

This section outlines details on feature tracking usage, optimization and verification steps.

3.1 Left Atrial Feature Tracking in Retrospective Gated CT

The task of LA feature tracking in retrospective gated CT images can be re-framed as non-rigid image registration. To track LA features, all later CT image frames need to be registered to the initial end-diastolic (ED) CT image. This provides displacement fields to deform the LA model from ED to all the later time frames in the cardiac cycle. Strains can then be calculated on the deformed model. In this study, we firstly optimized and applied the temporal sparse free form deformation (TSFFD) method [17] for LA feature tracking in retrospective gated CT.

Images were cropped to cover the LA body, PV ostia and LAA prior to feature tracking. This ensured that the images fit within computational memory constraints, and avoided spatial resolution downsampling which ensured sufficient image resolution of the LA was achieved.

3.2 Temporal Sparse Free Form Deformation Method

The TSFFD method was used as it is based upon the widely used free form deformation method, with additional sparsity and cyclicity constraints which encourage temporally and spatially smooth displacement fields that are appropriate for the application of cardiac motion measurement. It has previously been applied to LV feature tracking in retrospective gated CT. The TSFFD method performs nonrigid registration of all later time frames to the initial end-diastolic CT frame simultaneously using multiple levels of control points with increasing spatial resolution. Control point (CP) displacements are parametrized using cubic B-spline functions in both space and time which encourage spatially and temporally smooth displacement fields. The sparsity constraint encourages sparsely activated CPs.

3.3 TSFFD Optimisation

The workflow for the optimisation of the TSFFD image registration method is outlined in Figure 4. The sparsity weight (SW) and bending energy (BE) parameters, which parametrize the sparsity constraint and stiffness of deformation respectively, were optimized using a grid search through 63 different SW and BE combinations. The SW and BE values that were iterated through are displayed in Figure 5. The grid search was performed at a coarser and finer set of multi-level control point resolutions, which are outlined in Table 1.

Supplementary Table 1. Two sets of control point (CP) resolutions at which hyperparameters for the TSFFD method were searched through. A coarser and finer set of CP resolutions were tested.

Resolution	Level 1 [mm^3]	Level 2 [mm^3]	Level 3 [mm^3]	Level 4 [mm^3]
Coarse	5 x 5 x 5	10 x 10 x 10	20 x 20 x 20	40 x 40 x 40
Fine	2 x 2 x 2	5 x 5 x 5	10 x 10 x 10	20 x 20 x 20

The LA feature tracking accuracy was quantified using three error metrics based upon tracking LA features from end-diastole (ED) to end-systole (ES). The ED-to-ES registration was chosen since it involves the largest change of shape of the LA and will therefore generate the largest errors in LA feature tracking. Surface meshes and segmentations of the LA body at ED were deformed to ES using the TSFFD deformation field and compared with the ground truth surface meshes and segmentations created from the target image at ES.

Surface mesh-based and segmentation-based errors were calculated: the average surface distance (ASD) and directed Hausdorff distance (DHD) and the dice score coefficient (DSC). ASD is the mean normal distance between the tracked and ground truth meshes at ES, and DHD is the maximal minimum distance between points in the tracked and ground truth ES meshes. Lower values for ASD and DHD indicate more accurate LA feature tracking. DSC represents the overlap in LA body segmentation between the tracked and ground truth LA segmentations at ES. Higher values for DSC indicate more accurate LA feature tracking.

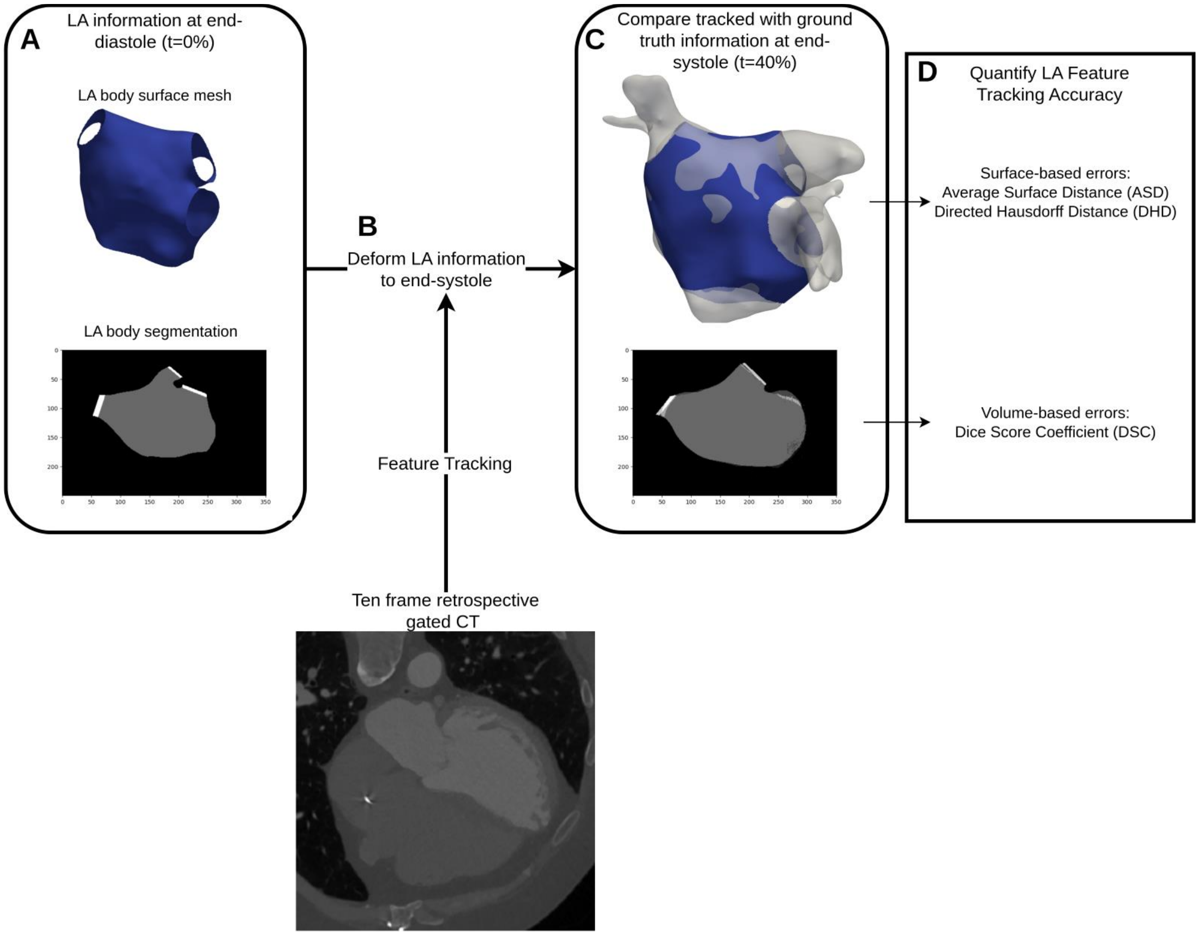

Supplementary Figure 4. Schematic that depicts quantification of LA feature tracking accuracy for optimization. **A:** Anatomical information of the LA body at the end-diastolic CT frame was captured using an endocardial surface mesh and segmentation. **B:** Surfaces and segmentations were deformed from end-diastole (t=0%) to end-systole (t=40%) using the deformation calculated from the feature tracking from the retrospective gated CT images. **C:** The tracked surface (in blue) was compared with the ground truth surface (in transparent grey) and the tracked segmentation (solid) was compared with the ground truth segmentation (transparent) end-systole. **D:** Differences in surfaces and segmentations at end-systole were quantified using surface-based errors (average surface distance and directed Hausdorff distance) and volume-based errors (dice score coefficient), respectively.

A combination of the ASD, DHD and DSC errors with equal weighting was used to identify the optimal TSFFD hyperparameter combination for LA feature tracking. To combine the error metrics, errors were first normalized by calculating the Z-score, which represents the number of standard deviations away from the mean across the cohort:

$$Z = \frac{x - \mu}{\sigma}$$

For each hyperparameter combination, the Z-scores of each error metric were combined to give the unified score,

$$S_{unified} = \frac{Z_{ASD} + Z_{DHD} + 1 - Z_{DSC}}{3}.$$

Lower values of $S_{unified}$ represent more accurate LA feature tracking across the cohort. Five-fold cross validation was used with 25 patient cases to select the optimal hyperparameter combination. This ensured the optimal hyperparameter combination was robust to which cases were used to calculate the LA feature tracking errors using $S_{unified}$. Results of the 5-fold cross validation used for the feature tracking optimization are shown in Figure 5. The hyperparameter combination which gave the lowest $S_{unified}$ score was SW = 0.0, BE = 1e-9. This combination gave an $S_{unified}$ score of –1.47.

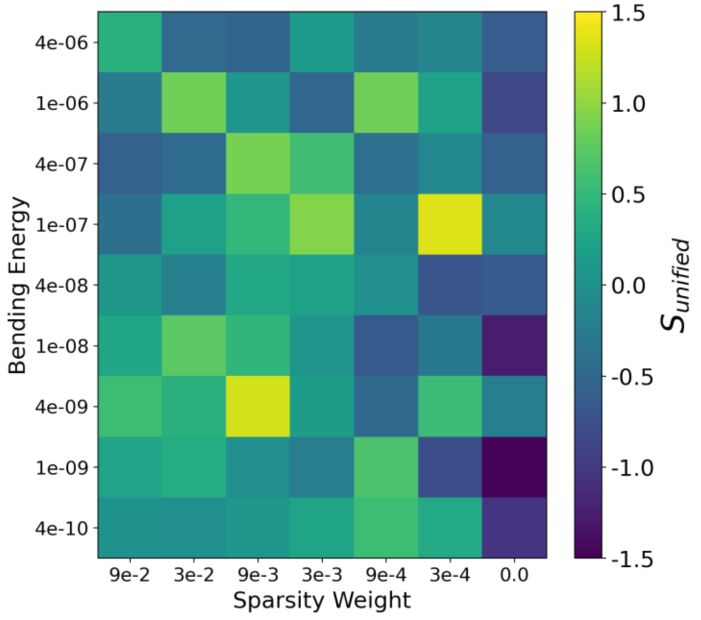

Supplementary Figure 5. Results of the feature tracking optimization using 5-fold cross validation for the Bending Energy and Sparsity Weight hyperparameters. Lower values of $S_{unified}$, indicated by darker blue shaded squares, represent more accurate LA feature tracking in the CT images.

A hold-out test set of 5 patient cases was used to evaluate the accuracy of the feature tracking on an unseen dataset. Error metrics for the test set are shown in Figure 6. The mean ± standard deviation of the test set errors were as follows, ASD: 0.52 ± 0.16 mm, DHD: 4.22 ± 2.32 mm, DSC: 95 ± 2 %.

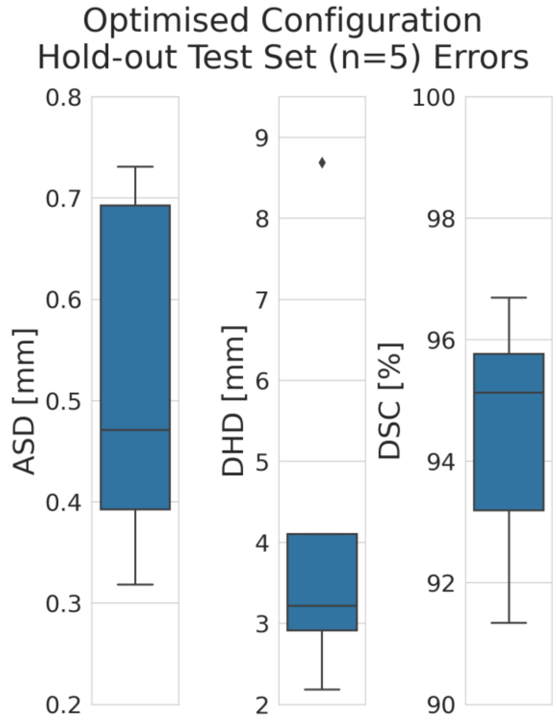

Supplementary Figure 6. Optimised TSFFD performance on a hold-out test set. Abbreviations: ASD – average surface distance; DHD – directed Hausdorff distance; DSC – dice score coefficient.

3.4 Feature Tracking Verification

Synthetically generated gated CT images with known LA deformation were used to verify the optimized feature tracking method. Calculated displacement fields using TSFFD for each patient were applied to the ED image to create a set of synthetic gated CT images. The synthetic CT images were then re-registered to yield inferred LA motion which was compared with the ground truth LA motion. This enabled the calculation of errors for material point tracking, and global and regional strain calculation. Synthetic CT images were created across the patient cohort, therefore our verification method captured inter-individual variability in atrial size and motion.

The Euclidean distance between inferred and ground truth positions for the LA endocardial surface vertex points was calculated using the ED to ES registration to give a measure of material point tracking error. The median and mean ± standard deviation of the root mean square error (RMSE) of the Euclidean distance between the inferred and ground truth positions were 0.4 mm and 0.6 ± 0.5 mm, respectively across the cohort. This suggests that our feature tracking method was able to predict vertex coordinates on the LA endocardial surface within roughly one voxel resolution.

The synthetic gated CT images enabled the comparison between inferred and ground truth strain measurements, as depicted in Figure 7. Ground truth (solid lines) and inferred (dotted lines) global and regional strain transients were compared for each case.

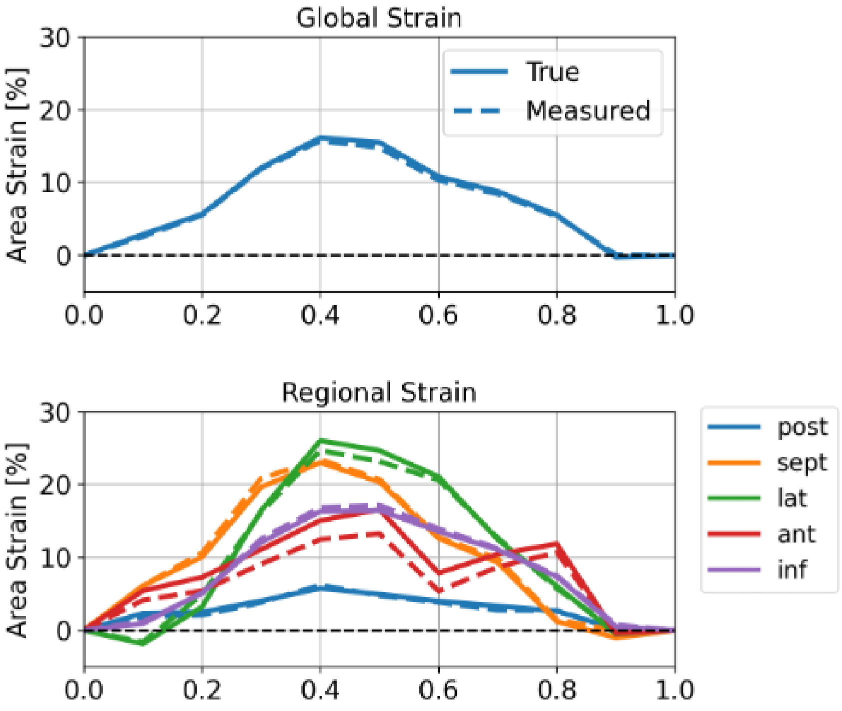

Supplementary Figure 7. Synthetic CT images with known LA deformation enabled the comparison of inferred and ground truth strain measurements. Shown above are inferred (dotted lines) and ground truth (solid line) strain transients for a sample HFrEF only case. The shape of the strain transients are similar – differences in strain transient shape can be quantified using the root mean square error (RMSE). Absolute differences in reservoir strain measurements can also be quantified.

Differences between the ground truth and inferred strain transients are described using the root mean square error (RMSE). The RMSE values across the cohort are outlined in Table 2 for global and regional strain transients. Absolute differences in reservoir strain measurements across the cohort are outlined in Table 3.

Supplementary Table 2. Statistics of root mean square error (RMSE) values between the ground truth and inferred strain transients. Statistics are given per patient group.

Patient Group	Strain Metric	Region	Mean ± SD [%]	Median [%]
HFrEF-only				
	Area Strain	Global	1.3 ± 1.5	0.5
		Posterior Wall	1.4 ± 1.0	0.9
		Septum	2.2 ± 2.1	1.6

		Lateral Wall	5.9 ± 5.7	3.2
		Anterior Wall	2.1 ± 1.3	1.7
		Inferior Wall	2.8 ± 3.6	1.5
	Fiber Strain	Global	0.9 ± 0.8	0.6
		Posterior Wall	1.2 ± 1.2	0.8
		Septum	2.1 ± 2.0	1.5
		Lateral Wall	3.2 ± 2.6	2.5
		Anterior Wall	1.6 ± 1.5	0.9
		Inferior Wall	1.1 ± 0.8	0.9
HFrEF+AF				
	Area Strain	Global	0.4 ± 0.4	0.3
		Posterior Wall	1.1 ± 0.8	0.8
		Septum	1.3 ± 0.6	1.4
		Lateral Wall	1.5 ± 0.8	1.6
		Anterior Wall	1.2 ± 0.6	0.9
		Inferior Wall	0.8 ± 0.4	0.7
	Fiber Strain	Global	0.4 ± 0.2	0.3
		Posterior Wall	0.8 ± 0.6	0.6

		Septum	1.2 ± 0	1.0
		Lateral Wall	1.1 ± 0.6	1.1
		Anterior Wall	0.9 ± 0.3	0.8
		Inferior Wall	0.6 ± 0.2	0.6

Supplementary Table 3. Statistics of absolute error in reservoir strain measurements between ground truth and inferred reservoir strains. Statistics are given per patient group.

Patient Group	Strain Metric	Region	Mean ± SD [%]	Median [%]
HFrEF-only				
	Area Strain	Global	2.0 ± 2.9	0.8
		Posterior Wall	1.4 ± 1.5	0.9
		Septum	2.8 ± 3.9	1.3
		Lateral Wall	5.9 ± 7.4	2.5
		Anterior Wall	2.4 ± 2.0	1.9
		Inferior Wall	4.1 ± 6.7	1.1
	Fiber Strain	Global	1.2 ± 1.4	0.5
		Posterior Wall	1.6 ± 1.9	0.6
		Septum	2.6 ± 3.2	1.6
		Lateral Wall	4.7 ± 5.9	1.9
		Anterior Wall	2.1 ± 1.96	1.5

		Inferior Wall	1.5 ± 1.2	1.0
HFrEF+AF				
	Area Strain	Global	0.4 ± 0.5	0.2
		Posterior Wall	2.1 ± 3.3	0.9
		Septum	1.2 ± 0.8	1.3
		Lateral Wall	1.7 ± 1.2	1.6
		Anterior Wall	1.1 ± 0	0.9
		Inferior Wall	0.8 ± 0.7	0.7
	Fiber Strain	Global	0.5 ± 0.4	0.3
		Posterior Wall	1.1 ± 1.2	0.6
		Septum	1.1 ± 1.1	0.4
		Lateral Wall	0.8 ± 0.6	0.7
		Anterior Wall	0.7 ± 0.7	0.3
		Inferior Wall	0.6 ± 0.5	0.6

3.5 Two-dimensional Feature Tracking

Two-dimensional (2D) global longitudinal strain (GLS) was calculated by extracting 2- and 4-chamber views from the RGCT images using manual landmarks, as shown in Figure 8. The coupled cross-hair rotation tool in CemrgApp was used to align imaging planes with the 2- and 4- chamber views. The TSFFD method was used to track LA features in the 2D images.

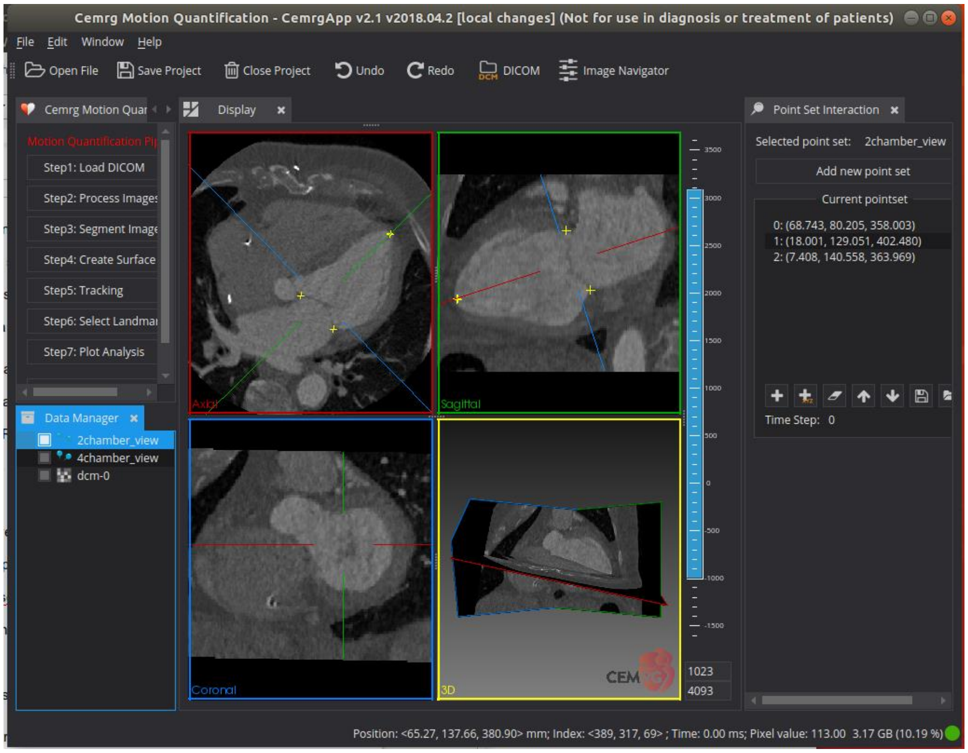

Supplementary Figure 8. Extraction of the 2- and 4-chamber views from RGCT images for 2D GLS strain calculation. Views were constructed using the coupled cross-hair rotation tool in CemrgApp, where 3 manual landmarks were chosen at the LV apex and MV to define each plane. Landmarks are shown as yellow cross-hairs. Top left: 4-chamber view. Top right: 2-chamber view.

3.6 Supplemental Videos

Visualization of the 3D LA feature tracking using retrospective gated CT images can be found in the four videos in the Supplements. Movies 1 and 2 display LA deformation measured using the optimised feature tracking throughout the cardiac cycle from two different views overlaid on the retrospective gated CT images. Movies 3 and 4 display LA deformation overlaid with area strain colourmaps, where red and blue colours correpond to expansion and contraction, respectively.

4 Strain Calculation

4.1 Area and Fiber Strain

The area and fiber strain metrics were used to quantify 3D LA deformation. This section outlines the equations used for calculating the strain metrics. All strains were calculated with respect to the initial LA shape at end-diastole (t=0%) which was timed to the R-wave on the ECG.

Area strain is defined as the percentage change of a local element on the LA endocardium surface. Specifically, the area strain of a local element at a later time t, $\epsilon_A(t)$, was defined as

$$\epsilon_A(t) = \frac{A(t) - A(t=0)}{A(t=0)} \times 100\%$$

Where $A(t)$ represents the element area at time t, and $A(t = 0)$ represents the initial element area at end-diastole. Positive area strain indicates endocardial area stretch and negative area strain indicates endocardial area contraction.

Endocardial fiber strain denotes deformation parallel to the local endocardial fiber direction. The calculation for fiber strain, ϵ_f, is described as follows. First, the green strain tensor, E , for each element was calculated from the deformation gradient, F , using

$$E = \frac{1}{2}F^T F - 1.$$

Fiber strain is calculated by first finding the green strain, ϵ_G, by projecting the green strain tensor onto the direction parallel to the element myofiber orientation, N :

$$\epsilon_G = N^T E N$$

Green strain was then converted to linear strain to calculate fibre strain, ϵ_f:

$$\epsilon_f = \left(\sqrt{2\epsilon_G + 1} - 1\right) \times 100\%$$

4.2 Difference between Strain Metrics

Area strain does not account for the direction of deformation, unlike fiber strain which measures deformation with respect to the local myofiber direction. Figure 9 depicts the differences between area and fiber strains using a sample of a LA endocardial surface.

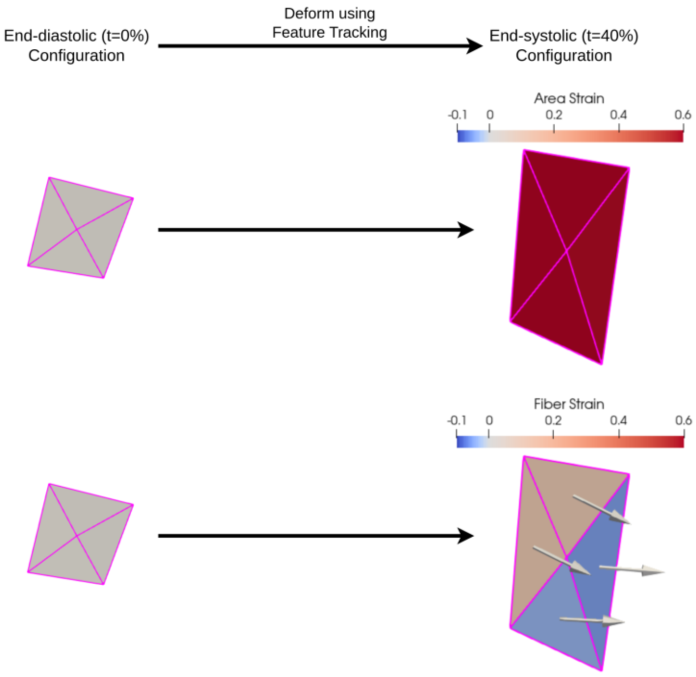

Supplementary Figure 9. Depiction of the different information that can be extracted from the area strain and fiber strain metrics. A subset of the left atrial endocardium surface is shown at its end-

diastolic (left) and end-systolic (right) configurations. Individual elements are outlined in pink. Area strain and fiber strain were both calculated, with red and blue colors representing positive and negative strain, respectively. The local myofiber orientation for each element is shown by the arrowed glyphs. Top row, right: Area strain is colored red for all elements which reflects the uniform increase in area. Bottom row, right: Fiber strain is shaded light red for two elements and light blue for the remaining elements since deformation along the fiber directions are increasing and decreasing, respectively.

4.3 Reservoir Strain Measurement

To avoid artefacts in strain measurements due to pacing leads in the gated CT imaging, the top 0.1% of elemental strains were omitted from the strain analysis when taking mean averages over elements for regional and global reservoir strains. This circumvented extreme unphysiological strains from using the feature tracking with imaging that included lead artefacts from CRT patients.

4.4 Two-dimensional Global Longitudinal Strain Measurement

2D GLS was measured in both the 2- and 4- chamber views using the percentage length changes of the LA body contour, excluding the MV, PVs and LAA. Final values for 2D GLS were taken using the mean strain value from both the 2- and 4- chamber views. Visualisations of LA body contours in the 4- chamber view is displayed in Figure 10.

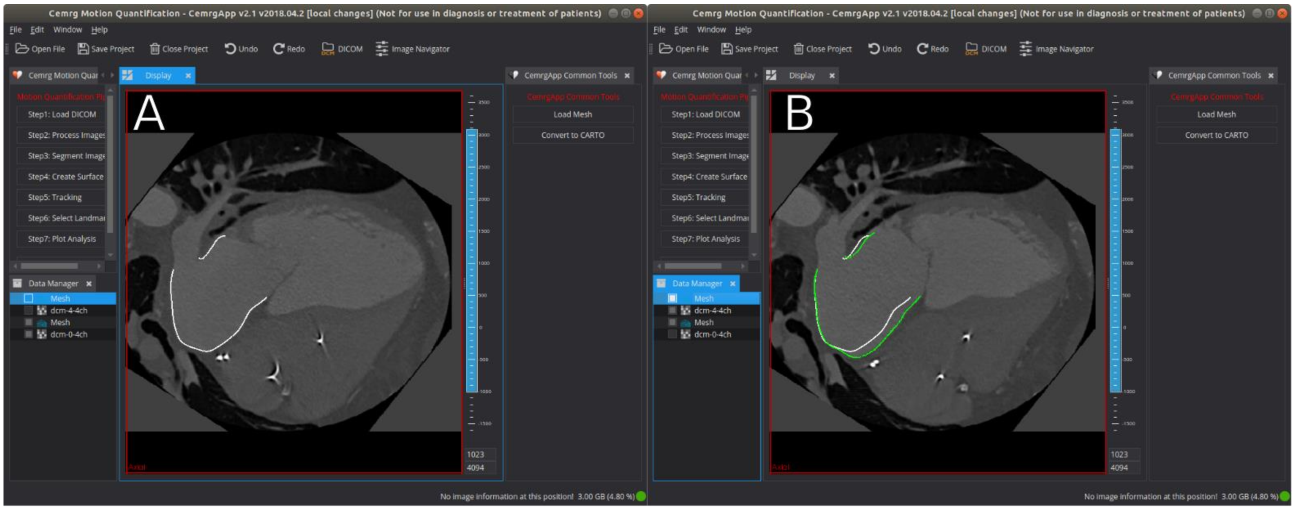

Supplementary Figure 10. 2D GLS calculated from the 4-chamber view for a sample case. A: End-diastolic (t=0%) frame and overlaid LA body contour, exlcuding the PVs, MV and LAA. B: End-systolic (t=40%) frame and overlaid tracked LA body contour at t=40% shown in green, which is larger than the original LA body contour, shown in white, from the end-diastolic frame.
